# Associations of wheezing phenotypes in the first 6 years of life with atopy, lung function and airway responsiveness in mid-childhood

**DOI:** 10.1136/thx.2007.093187

**Published:** 2008-08-04

**Authors:** J Henderson, R Granell, J Heron, A Sherriff, A Simpson, A Woodcock, D P Strachan, S O Shaheen, J A C Sterne

**Affiliations:** 1 Department of Community Based Medicine, University of Bristol, Bristol, UK; 2 Department of Social Medicine, University of Bristol, Bristol, UK; 3 North West Lung Centre, University of Manchester, Manchester, UK; 4 Department of Community Health Sciences, St George’s Hospital Medical School, London, UK; 5 London and Respiratory Epidemiology & Public Health Group, National Heart & Lung Institute, Imperial College, London, UK

## Abstract

**Background:**

Patterns of wheezing during early childhood may indicate differences in aetiology and prognosis of respiratory illnesses. Improved characterisation of wheezing phenotypes could lead to the identification of environmental influences on the development of asthma and airway diseases in predisposed individuals.

**Methods:**

Data collected on wheezing at seven time points from birth to 7 years from 6265 children in a longitudinal birth cohort (the ALSPAC study) were analysed. Latent class analysis was used to assign phenotypes based on patterns of wheezing. Measures of atopy, airway function (forced expiratory volume in 1 s (FEV_1_), mid forced expiratory flow (FEF_25-75_)) and bronchial responsiveness were made at 7–9 years of age.

**Results:**

Six phenotypes were identified. The strongest associations with atopy and airway responsiveness were found for intermediate onset (18 months) wheezing (OR for atopy 8.36, 95% CI 5.2 to 13.4; mean difference in dose response to methacholine 1.76, 95% CI 1.41 to 2.12 %FEV_1_ per μmol, compared with infrequent/never wheeze phenotype). Late onset wheezing (after 42 months) was also associated with atopy (OR 6.6, 95% CI 4.7 to 9.4) and airway responsiveness (mean difference 1.61, 95% CI 1.37 to 1.85 %FEV_1_ per μmol). Transient and prolonged early wheeze were not associated with atopy but were weakly associated with increased airway responsiveness and persistent wheeze had intermediate associations with these outcomes.

**Conclusions:**

The wheezing phenotypes most strongly associated with atopy and airway responsiveness were characterised by onset after age 18 months. This has potential implications for the timing of environmental influences on the initiation of atopic wheezing in early childhood.

Asthma is a complex heterogeneous disease comprising a number of discrete phenotypes, such that the term “asthma” has recently been called into question.^1^ Cohort studies followed to adulthood have reported that more severe childhood wheezing phenotypes are less likely to remit in later life.^2^^–^4 Pulmonary function abnormalities associated with persistent wheezing become established during early childhood and track to adult life, suggesting that early life exposures are critical in determining the onset and natural history of wheezing illnesses.^3^ ^5^ An improved understanding of these phenotypes is therefore of fundamental importance to studies of risk factors for asthma and wheezing illnesses in children.

In a seminal report based on the Tucson Children’s Respiratory Study, Martinez and colleagues proposed three patterns of wheezing during the first 6 years of life^6^ leading to the concepts of transient early wheezing in the first 3 years, non-atopic wheezing in the preschool years and IgE-mediated wheeze or asthma.^7^ Although these have served as useful models of wheezing phenotypes in early childhood, there is evidence that phenotypes diverge earlier than 3 years^8^ and a recent report described variations in immune responses within seemingly homogeneous phenotypes of IgE-mediated asthma.^9^ We used a novel symptom-driven approach to define wheezing phenotypes using repeat measurements of wheeze during the first 7 years of childhood in a large population-based birth cohort study, the Avon Longitudinal Study of Parents and Children (ALSPAC). We investigated associations of these phenotypes with physician-diagnosed asthma and objectively measured atopy and airway function at age 7–9 years.

## METHODS

### Participants

ALSPAC is a longitudinal population-based birth cohort study that recruited 14 541 pregnant women resident in Avon, UK with expected dates of delivery 1 April 1991 to 31 December 1992. There were 14 062 liveborn children. The study protocol has been described previously^10^ ^11^ and further details are shown on the ALSPAC website (http://www.alspac.bris.ac.uk).

### Data collection

At 6, 18, 30, 42, 54, 69 and 81 months after birth, study mothers were sent a self-completion questionnaire about the health of their child. They were asked to report the occurrence of 15 common symptoms, including wheezing, in the previous 12 months (6 months for the initial questionnaire) and, if present, whether they consulted a doctor. In a separate section they were asked whether in the past 12 months (6 months in the first questionnaire) their child had “wheezing with whistling on the chest when (s)he breathed”. At 91 months of age mothers were asked in a separate questionnaire to report if a physician had ever told them that their child had asthma.

The atopic status of the children was determined at 7–8 years of age by skin prick test responses to a panel of up to 12 common allergens including house dust mite (*Dermatophagoides pteronyssinus*), mixed grasses and cat (ALK; Abelló, Hoersholm, Denmark). Sensitisation to one of these three allergens has been shown to identify 95% of all sensitised children in this population.^12^ A positive response was defined as a mean weal diameter of ⩾2 mm with an absent response to negative control solution, and atopy was defined as a positive response to one or more of house dust mite, cat or grass pollen. Mothers were asked to report a personal history of asthma or allergy in a questionnaire administered during pregnancy.

At 8–9 years of age, lung function was measured by spirometry (Vitalograph 2120, Maids Moreton, UK) according to American Thoracic Society criteria.^13^ Flow-volume curves were reviewed by one respiratory physician (JH) to ensure adherence to standards, resulting in the rejection of 338 (4.6%) measurements and the correction of 883 (11.5%) where the automated programme had selected an inappropriate curve. Each variable (forced expiratory volume in 1 s (FEV_1_), forced vital capacity (FVC) and mid forced expiratory flow (FEF_25–75_)) was converted to sex-, age- and height-adjusted standard deviation units.^14^ Airway responsiveness to methacholine was measured using the method of Yan *et al*^15^ and expressed for each subject as the dose-response slope of FEV_1_ (percentage decline from baseline) per μmol methacholine.

### Statistical methods

Wheeze was defined as present if the response to either question about wheezing was “yes” and absent if the response to both was “no”. All other combinations were classed as missing (1.3%). As there were two levels of response to questions about wheeze at seven time points, there were 2^7^ = 128 different patterns of wheezing possible. Therefore, to derive phenotypes with similar wheezing patterns over time, we used latent class analysis. This is a statistical method for finding subtypes of related cases (latent classes) from multivariable categorical data (in this case, responses to wheezing questions across seven time points). Briefly, individuals were clustered into a number of discrete latent classes (phenotypes) on the basis of the pattern of responses to each of the wheezing questions. The latent class model aims to determine the minimum number of latent classes that describe the observed patterns of responses in the data.^16^ A full description of the latent class analysis used in this study and methods used to evaluate the best fitting model are shown in the online supplement. The posterior probability of each individual belonging to a particular phenotype was estimated and, from these data, the estimated prevalence of wheeze at each time point was calculated for each phenotype.

Children with complete reports of wheezing at all seven time points were included in the analyses. Logistic and linear regression was used to estimate associations of phenotype membership with physician-diagnosed asthma and objective measurements of atopy, lung function and bronchial responsiveness in mid childhood and with maternal self-reported asthma and allergy. As latent class analysis is robust to missing data and misclassification of data items such as faulty recall of wheezing episodes, we repeated all analyses in children who returned questionnaires at two or more time points.

All analyses were done using MPlus 4.1 software (Muthén & Muthén, Los Angeles, 2006).

## RESULTS

Of 11 678 children with reports of wheezing on at least two occasions, 6265 (54%) had complete data. The characteristics of the study population are shown in table 1. Children with complete data were less likely to come from socially deprived backgrounds and had lower prevalence of reported wheezing in early childhood than children with missing data.

**Table 1:** Characteristics of the study population with complete data on wheezing from birth to 81 months (n = 6265) compared with those with missing data

	Children with complete data on wheezing (n = 6265)		Children with 2–6 observations (n = 5413)		Children with 0–1 observations (n = 2384)	
	n/total	%	n/total	%	n/total	%
Girls	3029/6265	48	2623/5413	48	1138/2382	48
*Demographic data*						
Rented house	973/6143	16	1583/5116	31	943/1483	51
Mother not married	1041/6202	17	1427/5154	28	758/1864	41
Overcrowding	218/6088	4	404/5019	8	260/1771	15
One or more siblings	3289/6125	54	2862/5069	56	1043/1813	58
Low maternal education*	3496/6183	57	3531/4975	71	1038/1309	79
Teenage mother	89/6265	1	303/5413	6	263/2384	11
Mother manual occupation	793/5380	15	933/3851	24	284/868	33
Partner manual occupation	2158/5728	38	2112/4285	49	583/985	59
Prevalence of wheeze						
At 6 months	1506/6265	24	1338/4644	29	165/507	33
6 months	1506/6265	24	1338/4644	29	165/507	33
18 months	1645/6265	26	1334/4574	29	43/138	31
30 months	1336/6265	21	905/3671	25	21/54	39
42 months	1051/6265	17	705/3705	19	5/34	15
54 months	1128/6265	18	644/3122	21	7/23	30
69 months	938/6265	15	389/2327	17	1/9	11
81 months	852/6265	14	273/2127	13	4/11	36

*Low maternal education classified as “O” level or below (equivalent to school leaving certificate at 16 years in the UK).

Comparison of Bayesian information criteria (BIC; see online supplement) suggested that a model with six phenotypes provided the best fit (BIC from models with 3, 4, 5, 6 and 7 phenotypes were 34709, 34357, 34304, 34275 and 34285, respectively). Bootstrap likelihood ratio tests (BLRT) suggested a further improvement in fit comparing models with seven and eight phenotypes in cases with complete data only; analyses of data from children with at least two measures of wheezing from 6 to 81 months suggested—based on both BIC and BLRT—that a six-phenotype model provided the best fit. We therefore selected the more parsimonious solution and based further analyses on six-phenotype models for both datasets.

**Figure 1:**
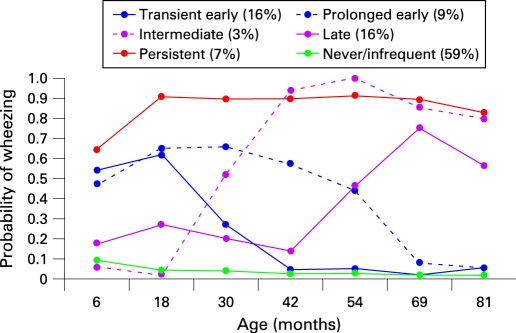
Estimated prevalence of wheezing at each time point from birth to 81 months for each of the six wheezing phenotypes identified by latent class analysis in 6265 children with complete data.

The estimated prevalence of wheezing at each time point in the six phenotypes is shown in fig 1. Hereafter, we describe the phenotypes as follows:

- 1: Never/infrequent wheeze (59.3% of children) had approximately 10% prevalence of wheezing at 6 months with declining prevalence of sporadic wheeze thereafter and included subjects (76.5% of this category) who never reported wheeze.
- 2: Transient early wheeze (16.3%) had 50–60% prevalence up to 18 months, declining to low prevalence from 42 months.
- 3: Prolonged early wheeze (8.9%) had a peak prevalence of around 65% at 30 months, declining to low prevalence from 69 months.
- 4: Intermediate onset wheeze (2.7%) had a low prevalence up to 18 months, rising rapidly to high prevalence from age 42 months.
- 5: Late onset wheeze (6.0%) had approximately 20% prevalence up to 42 months, rising to 50% or higher prevalence thereafter.
- 6: Persistent wheeze (6.9%) had 65% prevalence at 6 months with approximately 90% prevalence thereafter.

Patterns of wheezing were similar in 11 678 children with missing data (see fig E1 in online supplement).

### Association of wheezing phenotypes with atopy and parental asthma/allergy

Table 2 shows associations of wheezing phenotypes with skin test responses at age 7–8 years. Intermediate onset wheeze, late onset wheeze and persistent wheeze were strongly associated with atopy. Neither of the early wheezing phenotypes was associated with atopy or specific allergen sensitisation. Intermediate onset wheezing showed the strongest associations with atopy and with sensitisation to cat and house dust mite (*D pteronyssinus*) allergens. Late onset wheezing was also strongly associated with cat and house dust sensitisation and had the strongest association with grass pollen sensitisation.

**Table 2:** Associations of wheezing phenotype with asthma and atopy in 5397 children with complete data on wheezing and asthma at 7.5 years and 4331 children with skin prick test data at 7–8 years

Phenotype	Physician-diagnosed asthma		Atopy (any skin prick sensitivity)		Skin prick sensitivity to D pteronyssinus*		Skin prick sensitivity to cat*		Skin prick sensitivity to grass*	
	n/total (%)	OR (95% CI)	n/total (%)	OR (95% CI)	n/total (%)	OR (95% CI)	n/total (%)	OR (95% CI)	n/total (%)	OR (95% CI)
Transient early	79/931 (8.5%)	2.46 (1.48 to 4.09)	95/700 (13.6%)	0.8 (0.55 to 1.17)	52/707 (7.4%)	0.84 (0.51 to 1.38)	22/702 (3.1%)	0.86 (0.42 to 1.77)	52/706 (7.4%)	0.80 (0.48 to 1.32)
Prolonged early	183/509 (36.0%)	14.87 (10.68 to 20.71)	57/383 (14.9%)	0.89 (0.58 to 1.38)	32/387 (8.3%)	0.99 (0.56 to 1.72)	11/384 (2.9%)	0.77 (0.27 to 2.2)	25/386 (6.5%)	0.69 (0.36 to 1.35)
Intermediate	141/152 (92.8%)	325.75 (137.78 to 770.14)	71/114 (62.3%)	8.36 (5.24 to 13.36)	59/116 (50.9%)	11.12 (7.06 to 17.53)	42/115 (36.5%)	15.51 (9.45 to 25.46)	37/115 (32.2%)	4.83 (2.98 to 7.82)
Late	260/341 (76.2%)	84.60 (56.00 to 127.8)	145/257 (56.4%)	6.62 (4.67 to 9.39)	93/259 (35.9%)	5.97 (4.13 to 8.63)	69/257 (26.8%)	9.73 (6.32 to 14.98)	101/259 (39.0%)	6.54 (4.57 to 9.35)
Persistent	362/393 (92.1%)	307.93 (185.86 to 510.18)	123/296 (41.6%)	3.64 (2.76 to 4.81)	91/299 (30.4%)	4.66 (3.42 to 6.36)	66/296 (22.3%)	7.54 (5.17 to 11)	73/298 (24.5%)	3.27 (2.37 to 4.53)
Never/infrequent	126/3397 (3.7%	1 (reference)	419/2554 (16.4%)	1 (reference)	219/2580 (8.5%)	1 (reference)	92/2560 (3.6%)	1 (reference)	232/2575 (9.0%)	1 (reference)

*Mean weal diameter ⩾2 mm. CI, confidence interval; OR, odds ratio.

Maternal self-reported asthma and allergy were positively associated with all wheezing phenotypes compared with infrequent wheeze (table 3). The strongest association with both maternal phenotypes was seen with persistent wheeze.

**Table 3:** Associations of wheezing phenotypes with maternal self-reported asthma and allergy in 6133 children with complete data on wheezing and maternal history of asthma/allergy

Wheezing phenotype	Maternal asthma Odds ratio (95% CI)	Maternal allergy Odds ratio (95% CI)
Transient early	2.26 (1.62 to 3.15)	1.37 (1.11 to 1.71)
Prolonged early	2.68 (1.89 to 3.78)	1.79 (1.40 to 2.29)
Intermediate	3.54 (2.21 to 5.67)	1.53 (1.05 to 2.22)
Late	2.37 (1.58 to 3.57)	1.41 (1.06 to 1.88)
Persistent	4.17 (3.12 to 5.56)	2.09 (1.67 to 2.62)
Never/infrequent	1 (reference)	1 (reference)

### Association of wheezing phenotypes with asthma and lung function

All wheezing phenotypes were associated with physician-diagnosed asthma by age 91 months compared with the never/infrequent wheeze phenotype. The proportion of subjects with physician-diagnosed asthma and odds ratios (OR) (95% confidence interval (CI)) were as follows: transient early wheeze 8.5%, OR 2.5 (95% CI 1.5 to 4.1); prolonged early wheeze 36%, OR 14.9 (95% CI 10.7 to 20.7); intermediate onset wheeze 92.8%, OR 326 (95% CI 138 to 770); late onset wheeze 76.2%, OR 85 (95% CI 56 to 128); persistent wheeze 92.1%, OR 308 (95% CI 186 to 510).

Compared with the late onset phenotype, the intermediate onset phenotype was associated with a higher prevalence of doctor-diagnosed asthma (OR 3.9, 95% CI 1.1 to 13.2). Similarly, compared with the transient early phenotype, the prolonged early phenotype had an OR of asthma at 91 months of 6.0 (95% CI 3.5 to 10.3), reflecting the marked difference in prevalence of doctor-diagnosed asthma (9% and 36%, respectively) in the two groups.

Table 4 shows associations of wheezing phenotypes with lung function and airway responsiveness at 8–9 years of age. All phenotypes were associated with decrements of FEV_1_ and FEF_25–75_ and increased airway responsiveness compared with never/infrequent wheeze. The greatest decrements were associated with prolonged early, intermediate onset and persistent wheezing. Airway responsiveness was highest in the intermediate and late onset phenotypes.

**Table 4:** Associations of wheezing phenotype with lung function in 4448 children with complete data on wheezing and lung function measurements at 8–9 years and 2957 with airway responsiveness measurements at 8–9 years

Phenotype	FEV_1_ (l)			FEF_25–75_ (l/s)			Airway responsiveness*		
	Total	Mean (SD)	Mean difference (95% CI)	Total	Mean (SD)	Mean difference (95% CI)	Total	Mean (SD)	Mean difference (95% CI)
Transient early	724	−0.16 (0.91)	−0.29 (−0.37 to −0.21)	735	−0.12 (0.96)	−0.31 (−0.39 to −0.24)	481	0.03 (1.5)	0.29 (0.13 to 0.44)
Prolonged early	396	−0.17 (1.11)	−0.30 (−0.41 to −0.20)	402	−0.34 (0.95)	−0.54 (−0.64 to −0.44)	263	0.01 (1.5)	0.27 (0.07 to 0.47)
Intermediate	118	−0.4 (1.18)	−0.53 (−0.71 to −0.35)	120	−0.49 (1.17)	−0.69 (−0.87 to −0.51)	79	1.51 (1.7)	1.76 (1.41 to 2.12)
Late	265	−0.08 (0.98)	−0.21 (−0.33 to −0.08)	269	−0.21 (1.07)	−0.40 (−0.53 to −0.28)	176	1.36 (1.7)	1.61 (1.37 to 1.85)
Persistent	306	−0.27 (1.05)	−0.40 (−0.52 to −0.28)	310	−0.49 (1.12)	−0.68 (−0.80 to −0.57)	203	0.94 (1.8)	1.19 (0.96 to 1.42)
Never/infrequent	2639	0.13 (0.98)	0 (reference)	2679	0.2 (0.97)	0 (reference)	1755	−0.26 (1.6)	0 (reference)

*Mean of least squares dose-response slope (% decline in FEV_1_ per μmol methacholine).

CI, confidence interval; FEF_25–75_, mid forced expiratory flow; FEV_1_, forced expiratory volume in 1 s; SD, standard deviation.

Compared with late onset wheezing, the intermediate onset phenotype was associated with decrements of FEV_1_ and FEF_25–75_ of 0.33 (95% CI 0.10 to 0.55) and 0.28 (95% CI 0.05 to 0.52) standard deviations, respectively. There was also a decrement in mid-expiratory flow (mean difference for FEF_25–75_ −0.22 SD units (95% CI −0.34 to −0.11) in the prolonged early wheezing group compared with the transient early wheezing group.

### Never wheeze versus infrequent wheeze

As 2979/3896 subjects (76.5%) assigned to the never/infrequent wheeze phenotype had never reported wheeze, the associations with objective outcomes of these children were compared with the 917 subjects assigned to this group who reported at least one episode of wheeze. The never wheeze group had higher FEV_1_ (mean difference 0.14 SD units (95% CI 0.05 to 0.22)) and FEF_25–75_ (mean difference 0.18 SD units (95% CI 0.10 to 0.27)) and lower airway responsiveness (−0.30 percentage FEV_1_ per μmol methacholine (95% CI −0.13 to −0.47)) than those with at least one reported episode of wheeze. There were no differences in the prevalence of atopy or individual skin prick test responses between these two groups.

### Associations of wheezing phenotypes with other outcomes in children with missing data

The associations of wheezing phenotypes with maternal asthma and with later childhood outcomes in 11 678 children who returned at least two questionnaires on wheezing are shown in tables E3–E5 in the online supplement. These gave very similar results to the analyses based on children with complete data on wheezing between 6 and 81 months of age.

## DISCUSSION

Using data on reported wheezing collected at frequent intervals during the first 7 years in a large population-based birth cohort, we have identified six childhood wheezing phenotypes and quantified their associations with objective measures of atopy and lung function in mid-childhood. Two of these phenotypes have not been described previously. Prolonged early wheeze (around 9% of children) was characterised by wheezing from age 6 to 54 months with low prevalence from age 69 months onwards. It was not associated with aeroallergen sensitisation but was associated with increased airway responsiveness and lower lung function at ages 8–9 years compared with the never/infrequent wheeze phenotype. Intermediate onset wheeze (around 2.5% of children) had onset between ages 18 and 42 months. This phenotype was characterised by the strongest association with atopy (particularly skin prick sensitivity to *D pteronyssinus* and cat allergen), lower lung function and higher levels of airway responsiveness compared with the never/infrequent wheeze phenotype. Such associations (represented figuratively in table 5) may reveal differing aetiological or environmental influences on the inception of asthma in young children.

**Table 5:** Strength and direction of associations between derived phenotypes and clinical outcomes

Phenotype	Asthma	Atopy	FEV_1_	FEF_25–75_	AHR
Transient early	√	×	↓	↓	↑
Prolonged early	√√	×	↓	↓↓	↑
Intermediate onset	√√√√	√√	↓↓	↓↓	↑↑
Late onset	√√√	√√	↓	↓	↑↑
Persistent	√√√√	√	↓↓	↓↓	↑↑

The strength of association of each wheezing phenotype with each outcome is represented by the number of symbols (√, ↓ or ↑) with a cross (×) representing absence of association with that outcome.

AHR, airway hyper-responsiveness; FEF_25–75_, mid forced expiratory flow; FEV_1_, forced expiratory volume in 1 s.

Although the phenotypes identified here have similarities to previously reported patterns of early childhood wheezing, there were differences in their associations with objective outcomes. In the Tucson study, children with persistent and late onset wheeze had the strongest associations with atopy and those with persistent and transient early wheeze had the greatest decrements of lung function at age 6 and 11 years,^5^ with only persistent wheeze being positively associated with increased airway responsiveness at 11 years.^17^ Our results challenge these paradigms in that intermediate onset wheezing was most strongly associated with atopy and airway responsiveness in our study, although it should be noted that this pattern would have been included in the persistent wheeze phenotype as defined by the Tucson group. Persistent wheeze in the present study was less strongly associated with atopy than intermediate or late onset wheeze, but was associated with similar lung function deficits to intermediate onset wheeze, suggesting that persistent wheeze may represent a mixture of structural airway abnormalities associated with early onset wheezing and atopic wheeze that develops during early childhood.

In order to interpret the relevance of these findings to the heterogeneity of early childhood wheezing, it is necessary to appreciate the advantages and limitations of the latent class method used to identify the phenotypes in this study. As the term “latent classes” implies, these are not directly observed phenomena but were constructed post hoc on the basis of the pattern of responses to wheezing over a fixed number of observation periods. These methods are therefore not applicable to predicting the natural history of wheezing in individual subjects. Each child was assigned a probability of membership of each class based on their overall wheezing history. As shown in table E1 in the online supplement, some children had a high probability of membership of a single class while the assignment of others was less certain. This becomes increasingly evident in analyses including children with some missing observations of wheeze. Children with clear patterns of reported wheezing, such as those who always or never wheezed, had the highest probability of belonging to a single phenotype and therefore contributed the greatest weight to analyses of associations of these phenotypes with other outcomes. The advantage of the latent class approach is that the phenotypes were not constrained by prespecified notions of their number or nature; these were determined by the patterns observed in the data. Clear and interpretable associations of the different phenotypes with physician-diagnosed asthma and with objective measures of atopy and lung function at ages 7–9 years confirmed the utility of the approach. However, we acknowledge that the question of whether these phenotypes represent discrete pathophysiological entities cannot be resolved by the present analysis. For instance, it is conceivable that prolonged and transient early wheezing represents different severities of the same broad phenotype, with the more severe phenotype being associated with longer duration of wheeze and poorer prognosis. Future analyses of associations of these derived phenotypes with early life exposures that may contribute to their aetiology will help to address some of these issues.

An advantage of our study compared with previous cohort studies of the natural history of wheezing is its larger size, which allows investigation of associations of wheezing phenotypes with different measures of atopy and lung function, despite the fact that some phenotypes represented relatively small proportions of children. For comparison, the Tuscon Study reported on 826 children during the first 6 years,^6^ the Dunedin study on 613 subjects with complete respiratory data at 9–26 years,^3^ the Perth study of infant lung function reported outcomes at 11 years in 183 infants^18^ and a cohort of 2860 infants in Perth reported on asthma to age 6 years.^19^

There were also a number of limitations of our data. In common with most cohort studies,^20^ loss to follow-up was greater in children from more socially deprived backgrounds. Given known associations of social deprivation with early childhood wheezing,^21^ it is likely that children excluded because of missing data had a higher proportion of transient early wheezing than those included. We addressed this problem more comprehensively than previous cohort studies because we found similar results when we repeated latent class analyses using 11 678 children with two or more observations of wheeze.

The wheeze questionnaire that was devised for the ALSPAC study in 1991 contains similar questions to the ISAAC questionnaire now in common use.^22^ Care was taken to resolve discrepancies in responses to different questions about wheezing in the present study. However, it has been reported that parental-reported wheezing in early life is imprecise^23^ and correlates poorly with objective observations^24^ or with wheeze assessed by health professionals.^25^ Reassuringly, we found extremely strong associations (ORs up to 326) between wheezing phenotypes and physician-diagnosed asthma reported at age 91 months. Because we had up to seven observations of wheeze, and because the latent class approach allows for misclassification, lack of reliability of parental reporting of wheeze appears not to have been a problem in this study.

We did not have reliable data on treatment for wheeze in early life, although recent studies have suggested that treatment with inhaled corticosteroids in infancy does not alter the natural history of wheezing illnesses in children.^26^ ^27^ However, it is conceivable that treatment suppressed symptoms of wheeze completely in some subjects, which may have biased reporting towards those with more severe symptoms. Alternatively, suppression of symptoms by treatment with inhaled steroids may have contributed to misclassification of phenotypes that were based on parental-reported wheeze. As this is likely to have affected those phenotypes with the strongest associations with doctor-diagnosed asthma, we would have expected such an effect to attenuate differences between these and other phenotypic groups rather than to lead to spurious associations with objective outcomes. We plan to investigate markers of severity within phenotypes in future studies.

Our finding that the intermediate and late onset phenotypes had the strongest associations with atopy is consistent with a critical window of immunological responses^28^ during which environmental influences, such as allergens^29^ ^30^ or viral respiratory infections,^31^ interact with genetic variants in immune responsiveness to influence the risk of developing asthma and allergy.^32^ The association of late onset wheezing with grass pollen sensitisation may also represent a complex interplay of environmental exposures and genetic predisposition with the later onset of symptoms related to seasonal as opposed to ubiquitous allergen exposure.

Transient early wheezing has been associated with reduced lung function soon after birth, and there is evidence from several studies that such deficits are likely to improve partially in later childhood, although may continue to track below normal values.^5^ ^18^ ^33^ Early postnatal measurements were not available in our study but, based on this literature, it seems plausible that early decrements of lung function were associated with the three early onset wheezing phenotypes which were less strongly associated with atopy and airway responsiveness than later onset wheezing. Reduced lung function soon after birth is associated with asthma^34^ and airway responsiveness^35^ in later childhood. Our finding of mid-childhood lung function decrements in children with prolonged early and persistent wheezing could reflect persistence of developmental airway abnormalities, but is also consistent with allergenic or non-allergenic postnatal exposures aggravating existing structural airway abnormalities in subgroups of early onset wheeze. The importance of such decrements in mid-childhood is that, once established, they are likely to persist to adulthood.^3^ ^5^

In summary, the childhood wheezing phenotypes most strongly associated with atopy and airway responsiveness in our study were characterised by onset of wheezing after the age of 18 months. Wheezing onset soon after birth was not associated with atopy or airway responsiveness except when it persisted to later childhood. Persistent wheeze may represent a complex phenotype comprising different pathophysiological components encompassing early structural or functional airway changes modified by inflammatory processes during early childhood. Environmental influences on the initiation of atopic wheezing or which modify existing wheezing phenotypes are likely to have a major influence during the first years after birth. The search for modifiable factors that account for the rise in asthma and allergic diseases in industrialised countries^36^ should focus on interactions between genes and environment during this critical period. The availability of early environmental data in the ALSPAC cohort will enable these associations to be examined in relation to the phenotypes described here.

## References

[1] Anon A plea to abandon asthma as a disease concept. Lancet 2006;368:705 16935663 10.1016/S0140-6736(06)69257-X

[2] Phelan PD Robertson CF Olinsky A The Melbourne Asthma Study: 1964–1999. J Allergy Clin Immunol 2002;109:189–94 11842286 10.1067/mai.2002.120951

[3] Sears MR Greene JM Willan AR< A longitudinal, population-based, cohort study of childhood asthma followed to adulthood. N Engl J Med 2003;349:1414–22 14534334 10.1056/NEJMoa022363

[4] Jenkins MA Hopper JL Bowes G< Factors in childhood as predictors of asthma in adult life. BMJ 1994;309:90–3 8038673 10.1136/bmj.309.6947.90 PMC2540556

[5] Morgan WJ Stern DA Sherrill DL< Outcome of asthma and wheezing in the first 6 years of life: follow-up through adolescence. Am J Respir Crit Care Med 2005;172:1253–8 16109980 10.1164/rccm.200504-525OC PMC2718414

[6] Martinez FD Wright AL Taussig LM< Asthma and wheezing in the first six years of life. The Group Health Medical Associates. N Engl J Med 1995;332:133–8 7800004 10.1056/NEJM199501193320301

[7] Stein RT Martinez FD Asthma phenotypes in childhood: lessons from an epidemiological approach. Paediatr Respir Rev 2004;5:155–61 15135126 10.1016/j.prrv.2004.01.007

[8] Sherriff A Peters TJ Henderson J Strachan D, ALSPAC Study Team Avon Longitudinal Study of Parents and Children. Risk factor associations with wheezing patterns in children followed longitudinally from birth to 3(1/2) years. Int J Epidemiol 2001;30:1473–84 11821366 10.1093/ije/30.6.1473

[9] Heaton T Rowe J Turner S< An immunoepidemiological approach to asthma: identification of in-vitro T-cell response patterns associated with different wheezing phenotypes in children. Lancet 2005;365:142–9 15639296 10.1016/S0140-6736(05)17704-6

[10] Golding J Pembrey M Jones R ALSPAC—the Avon Longitudinal Study of Parents and Children. I. Study methodology. Paediatr Perinat Epidemiol 2001;15:74–87 11237119 10.1046/j.1365-3016.2001.00325.x

[11] Pembrey M The Avon Longitudinal Study of Parents and Children (ALSPAC): a resource for genetic epidemiology. Eur J Endocrinol 2004;151 (Suppl 3):U125–9 15554897 10.1530/eje.0.151u125

[12] Roberts G Peckitt C Northstone K< Relationship between aeroallergen and food allergen sensitization in childhood. Clin Exp Allergy 2005;35:933–40 16008681 10.1111/j.1365-2222.2005.02280.x

[13] American Thoracic Society Standardization of spirometry: 1994 update. Am J Respir Crit Care Med 1995;152:1107–36 7663792 10.1164/ajrccm.152.3.7663792

[14] Chinn S Rona RJ Height and age adjustment for cross sectional studies of lung function in children aged 6–11 years. Thorax 1992;47:707–14 1440464 10.1136/thx.47.9.707 PMC474803

[15] Yan K Salome C Woolcock AJ Rapid method for measurement of bronchial responsiveness. Thorax 1983;38:760–5 6648855 10.1136/thx.38.10.760 PMC459653

[16] Rabe-Hesketh S Skrondal A Classical latent variable models for medical research. Stat Methods Med Res 2008;17:5–32 17855748 10.1177/0962280207081236

[17] Stein RT Holberg CJ Morgan WJ< Peak flow variability, methacholine responsiveness and atopy as markers for detecting different wheezing phenotypes in childhood. Thorax 1997;52:946–52 9487341 10.1136/thx.52.11.946 PMC1758449

[18] Turner SW Palmer LJ Rye PJ< Infants with flow limitation at 4 weeks: outcome at 6 and 11 years. Am J Respir Crit Care Med 2002;165:1294–8 11991882 10.1164/rccm.200110-018OC

[19] Oddy WH Holt PG Sly PD< Association between breast feeding and asthma in 6 year old children: findings of a prospective birth cohort study. BMJ 1999;319:815–9 10496824 10.1136/bmj.319.7213.815 PMC314207

[20] Young AF Powers JR Bell SL Attrition in longitudinal studies: who do you lose? Aust NZ J Public Health 2006;30:353–61 10.1111/j.1467-842x.2006.tb00849.x 16956166

[21] Baker D Henderson J Differences between infants and adults in the social aetiology of wheeze. The ALSPAC Study Team. Avon Longitudinal Study of Pregnancy and Childhood. J Epidemiol Community Health 1999;53:636–42 10616676 10.1136/jech.53.10.636 PMC1756779

[22] Asher MI Keil U Anderson HR< International Study of Asthma and Allergies in Childhood (ISAAC): rationale and methods. Eur Respir J 1995;8:483–91 7789502 10.1183/09031936.95.08030483

[23] Elphick HE Sherlock P Foxall G< Survey of respiratory sounds in infants. Arch Dis Child 2001;84:35–9 11124781 10.1136/adc.84.1.35 PMC1718612

[24] Elphick HE Ritson S Rodgers H< When a “wheeze” is not a wheeze: acoustic analysis of breath sounds in infants. Eur Respir J 2000;16:593–7 11106197 10.1034/j.1399-3003.2000.16d04.x

[25] Lowe LA Simpson A Woodcock A< Wheeze phenotypes and lung function in preschool children. Am J Respir Crit Care Med 2005;171:231–7 15502115 10.1164/rccm.200406-695OC

[26] Guilbert TW Morgan WJ Zeiger RS< Long-term inhaled corticosteroids in preschool children at high risk for asthma. N Engl J Med 2006;354:1985–97 16687711 10.1056/NEJMoa051378

[27] Murray CS Woodcock A Langley SJ< Secondary prevention of asthma by the use of Inhaled Fluticasone propionate in Wheezy INfants (IFWIN): double-blind, randomised, controlled study. Lancet 2006;368:754–62 16935686 10.1016/S0140-6736(06)69285-4

[28] van der Velden V Laan MP Baert MR< Selective development of a strong Th2 cytokine profile in high-risk children who develop atopy: risk factors and regulatory role of IFN-gamma, IL-4 and IL-10. Clin Exp Allergy 2001;31:997–1006 11467989 10.1046/j.1365-2222.2001.01176.x

[29] Sporik R Holgate ST Platts-Mills TA< Exposure to house-dust mite allergen (Der p I) and the development of asthma in childhood. A prospective study. N Engl J Med 1990;323:502–7 2377175 10.1056/NEJM199008233230802

[30] Brussee JE Smit HA van Strien RT< Allergen exposure in infancy and the development of sensitization, wheeze, and asthma at 4 years. J Allergy Clin Immunol 2005;115:946–52 15867850 10.1016/j.jaci.2005.02.035

[31] Singh AM Moore PE Gern JE< Bronchiolitis to asthma: a review and call for studies of gene-virus interactions in asthma causation. Am J Respir Crit Care Med 2007;175:108–19 17053206 10.1164/rccm.200603-435PP

[32] Hoffjan S Nicolae D Ostrovnaya I< Gene-environment interaction effects on the development of immune responses in the 1st year of life. Am J Hum Genet 2005;76:696–704 15726497 10.1086/429418 PMC1199307

[33] Lau S Illi S Sommerfeld C< Transient early wheeze is not associated with impaired lung function in 7-yr-old children. Eur Respir J 2003;21:834–41 12765430 10.1183/09031936.03.00037203

[34] Haland G Carlsen KC Sandvik L< Reduced lung function at birth and the risk of asthma at 10 years of age. N Engl J Med 2006;355:1682–9 17050892 10.1056/NEJMoa052885

[35] Turner SW Palmer LJ Rye PJ< The relationship between infant airway function, childhood airway responsiveness, and asthma. Am J Respir Crit Care Med 2004;169:921–7 14764431 10.1164/rccm.200307-891OC

[36] International Study of Asthma and Allergies in Childhood (ISAAC) Steering Committee Worldwide variation in prevalence of symptoms of asthma, allergic rhinoconjunctivitis, and atopic eczema: ISAAC. Lancet 1998;351:1225–32 9643741

